# Impact of Gelatin Supplemented with Gum Arabic, Tween 20, and β-Cyclodextrin on the Microencapsulation of Turkish Oregano Extract

**DOI:** 10.3390/molecules24010176

**Published:** 2019-01-04

**Authors:** Juste Baranauskaite, Dalia M. Kopustinskiene, Jurga Bernatoniene

**Affiliations:** 1 Institute of Pharmaceutical Technologies, Medical Academy, Lithuanian University of Health Sciences, Sukileliu pr. 13, LT-50161 Kaunas, Lithuania; Juste.Baranauskaite@lsmuni.lt (J.B.); DaliaMarija.Kopustinskiene@lsmuni.lt (D.M.K.); 2 Department of Analytical and Toxicological Chemistry, Lithuanian University of Health Sciences, Medical Academy, A. Mickeviciaus g. 9, LT-44307 Kaunas, Lithuania; 3 Department of Drug Technology and Social Pharmacy, Medical Academy, Lithuanian University of Health Sciences, Sukileliu pr. 13-527, LT-50161 Kaunas, Lithuania

**Keywords:** microencapsulation, spray-drying, Turkish oregano, wall material, gelatin

## Abstract

Microencapsulation protects core materials from deteriorating due to environmental conditions, such as moisture or oxidation, and improves the bioavailability of active compounds, allowing one to make solid formulations from oils and increase their solubility. Wall and core material properties determine the microencapsulation efficiency and the best results are achieved when a wall material mixture is used to prepare the microcapsules. In this work, we optimized the wall material composition (gelatin supplemented with gum Arabic, Tween 20, and β-cyclodextrin) of Turkish oregano microcapsules prepared by spray-drying technology to increase the product yield, the encapsulation efficiency, and to achieve narrower particle size distribution. When the wall material solution contained 10 g of gelatin, 7.5 g of gum Arabic, 1.99 g of Tween 20, 1.98 g of β-cyclodextrin, and 20 g of ethanolic oregano extract, the encapsulation efficiency of oregano’s active compounds, rosmarinic acid and carvacrol, were 96.7% and 99.8%, respectively, and the product yield was 85.63%. The physicochemical properties, microscopic morphology, and in vitro release of the prepared microcapsules were characterized in the study. The use of gelatin as the main coating material, in supplementation with gum Arabic, Tween 20, and β-cyclodextrin, not only improved the encapsulation efficiency, but also increased the in vitro release of both main active compounds of Turkish oregano extract—rosmarinic acid and carvacrol.

## 1. Introduction

Turkish oregano (*Origanum onites* L.) (further referred as oregano) is an aromatic plant widely cultivated in Mediterranean countries and mainly used as a herb. Furthermore, oregano’s active compounds possess a variety of biological activities [1,2,3,4]. Oregano phenolic compounds have been shown to act as antibacterial and antifungal agents [3,4]. The antispasmodic, analgetic, diaphoretic, carminative, as well as antioxidant, antibacterial, antifungal activities of oregano essential oils have been extensively studied [2] and carvacrol has been identified as the main active compound responsible for these properties [1].

Solid formulations from active oregano compounds can be prepared using microencapsulation which helps to mask unpleasant odor or taste, to protect substances from moisture or oxidation, to alter solubility, and to prevent evaporation and incompatibilities [5]. Spray-drying encapsulation has been used for oregano essential oils [6,7], however, it has not been successfully applied to both essential oils and phenolic compounds yet. During our initial studies of microcapsules containing Turkish oregano extract, we identified the problems to obtain the higher product yield, the higher encapsulation efficiency, and narrower particle size distribution [8]. Maltodextrin and gum Arabic at the ratio 8.74:1.26 were used as the wall material in the initial studies, however, at low wall material proportions, it was not enough to fully cover the core with active compounds, resulting in low encapsulation efficiency (37.37% for rosmarinic acid and 7.75% for carvacrol) [8]. Furthermore, the relative span factor was 13.2, indicating a relatively wide particle size distribution [8]. According to Jafari et al. [9], microencapsulation efficiency largely depends on the properties of the wall and core materials. Furthermore, a single encapsulating matrix cannot fulfill all required characteristics [10], therefore mixtures of carbohydrates with proteins and polysaccharides at different proportions are used [10].

Gelatin, a derivative of collagen, is the protein most commonly used for encapsulation because of its ability to form a film and a gel. Gelatin is biocompatible, edible, biodegradable, and soluble at the body temperature and therefore it is an ideal material for food and pharmaceutical applications [11,12]. It is an amphoteric protein and is positively charged below its isoelectric point [11]. The main advantages of gelatin are its low toxicity [13], which has been proven clinically, and its ability to preserve the bioactivity of the therapeutic agent to be delivered in vivo [12]. Gelatin is a good choice as a wall material due to its good properties of emulsification, film-formation, water-solubility, high stabilizing activity, and a tendency to form a fine, dense network [14]. Repeated amino acid sequences are regularly required for the formation of the triple-helix structure, which is characteristic of the gelatin structure that is responsible for its ability to form gel. The segments in a triple-helix base form cross-linking in a three-dimensional network, which confers mechanical stability to the system at concentrations as low as 1% (0.01 g/g). It is possible to correlate emulsion stability with the system’s viscoelastic properties, since formation of a gel network within the continuous phase will slow down the movement of droplets due to gravity or Brownian motion. In general, increasing the viscosity of the emulsion aqueous phase may also provide better emulsion stability. The rheological properties of emulsions are mainly affected by quality (pH, ionic strength), availability of the solvent, and polymers present in the continuous aqueous phase [15,16]. In addition, gelatin has been successfully used as a wall material for both mixtures of ethanolic solution and volatile aroma during spray-drying [17]. Gum Arabic, a natural hydrocolloid and a highly branched arabinogalactan-protein, produces low-viscosity solutions at high concentrations, has excellent emulsifying properties, provides good volatiles retention during the drying process, and has the ability to create a strong protective film around the oil droplet [18]. β-cyclodextrin, a cyclic oligosaccharide, permits the encapsulation of essential oils [19,20,21], demonstrates the inclusion of major component molecules in the wall materials, and has fast release kinetics [21]. The surfactant Tween 20 helps to encapsulate hydrophobic compounds and readily forms emulsions with ethanol.

The objectives of the present work were to optimize the wall material composition (gelatin supplemented with gum Arabic, Tween 20, and β-cyclodextrin) of Turkish oregano microcapsules prepared by spray-drying to maximize the product yield and the encapsulation efficiency, to achieve narrower particle size distribution, to characterize the physicochemical properties and the microscopic morphology of the resultant microcapsules, and to study the release of the main oregano active compounds, rosmarinic acid and carvacrol, from the resultant microcapsules in vitro.

## 2. Materials and Methods

### 2.1. Materials

Dried *Origanum onites* L. herb was obtained from “İnanTarım ECO DAB’’, Turkey. Voucher specimens (No. L170711) have been deposited at the Herbarium of the Department of Drug Technology and Social Pharmacy, Lithuanian University of Health Sciences. Carvacrol (>98%), HPLC eluent acetic acid (99.8%), as well as wall material gum arabic and β-Cyclodextrin were purchased from Sigma-Aldrich (St. Louis, MO, USA). Tween 20 was purchased from Scharlau Chemie S.A. (Barcelona, Spain) and bovine Gelatin bloom value 100 was purschased from Gelita AG. (Eberbach, Germany). HPLC eluent methanol was from Carl Roth GmbH (Karlsruhe, Germany) and rosmarinic acid (>98%) was from ChromaDex (Santa Ana, TX, USA). Extraction solvent ethanol (96% *v/v*) was purchased from Vilniaus degtinė (Vilnius, Lithuania). The purified water used in HPLC and for sample preparation was produced with a Millipore Super Purity Water System (Sigma-Aldrich Corp., St.Louis, MO, USA).

### 2.2. Preparation of Oregano Ethanolic Extract

Prior to the extract preparation, the oregano herb was grounded in a cross beater mill IKA A11 Basic Grinder (IKA Works, Guanghou, China) and sieved using vibratory sieve shaker AS 200 basic (Retch, UK) equipped with a 125 µm sieve. Powdered material (100 g) was then extracted with 1000 mL of 90% (*v/v*) ethanol in a round bottom flask by heat-reflux extraction performed in a water bath Memmert WNB7 (Memmert GmbH & Co. KG, Schwabach, Germany) at 95 °C for 4 h. These conditions were determined as the best for the extraction of main active compounds of Turkish oregano in our previous study [22]. Prepared extract was filtered using vacuum filter. 

### 2.3. Preparation of Emulsion for Spray-Drying

The emulsion for the spray-drying consisted of the solution of gelatin, gum Arabic, and cyclodextrin (wall material), and oregano ethanolic extract with different ratios of Tween 20. The wall material solution was prepared by hydrating required amounts of gelatin, gum Arabic, and cyclodextrin in purified water dissolving them at 25 °C using magnetic stirrer hotplates (Heidolph MR, Germany) for 24 h. The emulsion for spray-drying (mixture of wall material solution and oregano ethanolic extract) was homogenized using magnetic stirrer for 1 h at 25 °C. The optimal ratio of wall material solution and ethanolic extract (wall material solution:oregano ethanolic extract) was obtained from previous studies [8]. In all experiments, 20 g of oregano ethanolic extract was used [8]. The wall material concentration in the emulsion was optimized in experimental design.

### 2.4. Experimental Design

The optimal wall material composition was determined in experimental design. The effects of four independent variables: amount of gelatin (A: 2–10 g), gum Arabic (B: 0–7.5 g), Tween 20 (C: 0–2 g), and β-cyclodextrin (D: 0–2 g) on two response variables, encapsulation efficiency of rosmarinic acid and encapsulation efficiency of carvacrol, were evaluated using D-optimal design. The ranges of parameters for the present investigation were determined from the preliminary experiments. Design-Expert^®^ (version 9.0.4.01, Stat-Ease Inc., Minneapolis, MN, USA) was used for regression analysis, the optimization procedure, and to select the best-fitting models. The variables and their levels used in the design are shown in Table 1. 

The design led to 30 combinations, including four replications at the central point. The analysis of variance (ANOVA) tables were generated and the effect and regression coefficients of individual linear models and the relationships between the variables were determined. The significance of all terms in the polynomial was judged statistically by computing the F value at the probability *p* < 0.05. Optimization of the fitted polynomials was done using numerical optimization and desirability function. The optimum conditions were verified by conducting experiments at the conditions determined. Responses were monitored and the results were compared with model prediction. The data were visualized by using Origin Pro 9.5 program (OriginLab Corporation, Northampton, MA, USA).

### 2.5. Microscopic Analysis

The determination of the average emulsion droplet size and emulsion type were made by optical microscopy using a BMS 739960 optical microscope equipped with a digital USB camera (Breukhoven, The Netherlands) for image processing. The diameter of 500 droplets in each sample was measured at 400× magnification using BMS software. The type of technologically optimized emulsion was confirmed by dyeing: the water soluble methylene blue was added to the continuous phase; the oil soluble Sudan II was added to the dispersive phase. A drop of the aqueous 5% solution of dyed emulsion was placed on a microscope slide and gently covered with a cover slip, the microscopic image was evaluated at 100× magnification using BMS software.

### 2.6. Determination of Emulsion Viscosity 

Viscosity of the prepared dispersions for the spray-drying was measured using the CAP-2000 Brookfield viscometer (Brookfield Engineering Laboratories, AMETEK Brookfield, IL, USA). The test sample was poured into a clean and dry 150 mL beaker and the viscosity of the test sample was measured during the standard operating procedure using 100 rpm spindle. The measurement time was 30 s at the temperature of 25 ± 2 °C.

### 2.7. Evaluation of Creaming

Samples of emulsions (1.5 g) were placed in a centrifuge tube and centrifuged at 3000 rpm for 5 min. The opaque region at the top had a lower density than the surrounding aqueous phase and therefore moved upwards due to centrifugal force. The extent of creaming was assessed by measuring the weight of the clear aqueous dispersion at the bottom and calculated using the following equation:

(1) $$\mathrm{CI}\;\left(\%\right)=\frac{\;a\times 100}{m},\;\;$$

where *a* is the amount of aqueous dispersion (g) and *m* is the mass of the sample (g).

### 2.8. Microcapsule Preparation 

The powders were obtained using a Buchi B-291 Mini Spray-Dryer (Flawil, Switzerland) operating with counter-current airflow. The equipment operated under the vacuum of 6 mBar and aspiration 100% in all experiments. The spray drying conditions depended on the previous studies optimal results conditions [8]. The inlet temperature was 170 °C and the feed flow rate was 40 mL/min, which was equivalent to 6% of the equipment’s full pumping capacity. The outlet temperatures depended on the inlet ones and were changing during the spray-drying process, when the inlet temperature was 170 °C the outlet ones ranged between 55–65 °C, outlet temperature has no significant data changes according the process. Outlet temperatures were the lowest at the beginning of spray-drying and gradually increased as the process continued.

### 2.9. Spray-Drying Product Yield and Microencapsulation Efficiency

Product yield means the percentage of encapsulated samples that the process actually yields in comparison to the theoretical yield:

(2) $$\mathrm{Yield}\left(\%\right)=\frac{\mathrm{Weight}\;\mathrm{of}\;\mathrm{obtained}\;\mathrm{microparticles}}{\mathrm{Weight}\;\mathrm{of}\;\mathrm{dry}\;\mathrm{compounds}\;\mathrm{in}\;\mathrm{mixture}\;\mathrm{before}\;\mathrm{spray}-\mathrm{drying}}\times 100$$

Weight of dry compounds consisted of equivalent amounts of wall materials (gelatin, gum Arabic, and β-cyclodextrin) and dry residue of oregano ethanolic extract (core).

Encapsulation efficiency, also known as active retention, is defined as the ratio of the concentration of encapsulated active ingredient (practical load) to its initial concentration at the beginning of the encapsulation process (theoretical load). It was calculated by the equations adopted from Panda et al. [23]:

(3) $$\mathrm{EE}\left(\%\right)=\frac{\mathrm{Practical}\;\mathrm{load}}{\mathrm{Theoretical}\;\mathrm{load}}\times 100$$

(4) $$\mathrm{Theoretical}\;\mathrm{load}\left(\%\right)=\frac{\mathrm{Total}\;\mathrm{drug}\;\left(\mathrm{active}\;\mathrm{compound}\right)}{\mathrm{Total}\;\mathrm{drug}\;\left(\mathrm{dry}\;\mathrm{residue}\right)+\mathrm{Total}\;\mathrm{excipients}\;\left(\mathrm{wall}\;\mathrm{material}\right)}\times 100$$

(5) $$\mathrm{Practical}\;\mathrm{load}\left(\%\right)=\frac{\mathrm{Weight}\;\mathrm{of}\;\mathrm{drug}\;\left(\mathrm{active}\;\mathrm{compound}\right)\mathrm{in}\;\mathrm{microparticles}}{\mathrm{Weight}\;\mathrm{of}\;\mathrm{microparticles}}\times 100$$

The monitored active compounds were rosmarinic acid and carvacrol. Quantitative estimation of rosmarinic acid and carvacrol in prepared microparticles was carried out using HPLC. One hundred mg of microcapsules were dissolved in 10 mL of mixture composed of ethanol 96% (*v/v*) and methanol at ratio 1:1. The solution was sonicated at 45 °C for 45 min in ultrasound bath (Memmert WNB7 water bath, Memmert GmbH & Co. KG, Schwabach, Germany), then samples were filtered through a 0.22 μm membrane filter and analyzed as described below.

For determination of rosmarinic acid, the mobile phase was composed of the solvent A (methanol) and the solvent B (0.5% (*v/v*) acetic acid in water). The following linear gradient elution profile was used: 95% A/5% B—0 min, 40% A/60% B—40 min, 10% A/90% B—41–55 min, 95% A/5% B—56 min. The flow rate was 1 mL/min and injection volume was 10 μL. The effluent was determined at a wavelength of 329 nm. The quantification has been carried out by the external standard method. The linear calibration curve was made (R^2^ = 0.999918), and the peak areas were used for quantification [8].

For determination of carvacrol, an ACE 5 C18 250 × 4.6 mm column (Advanced Chromatography Technologies, Aberdeen, Scotland) was used. The mobile phase was composed of methanol and water (60/40, *v/v*). The flow rate was 0.6 mL/min and injection volume was 10 μL. The absorption was measured at 275 nm. The quantification has been carried out by the external standard method. The calibration curve was made (R^2^ = 0.999751) [8].

### 2.10. Moisture Content

The spray-dried microencapsulates were analyzed for moisture content by estimating the powder’s weight loss after oven drying at 105 °C until a constant weight was obtained.

### 2.11. Flowability 

Carr index and Hausner ratio are quality control parameters for microcapsules which evaluate the flow properties of the powders [24].

Bulk and tapped volumes (*V_0_* and *V_tapped_*) were measured by the method from Pharmacopoeia (Ph. Eur., USP) using the density tester (SVM 102, Erweka, Germany). Determined values were then used for the calculation of the Hausner ratio and Carr index:

(6) $$\mathrm{Hausner}\;\mathrm{ratio}\;\left(\mathrm{HR}\right)=V_{0}/V_{tapped}$$

(7) $$\mathrm{Carr}\;\mathrm{index}\;\left(\mathrm{CI}\right)=\frac{100\times \left(V_{0}-V_{tapped}\right)}{V_{0}}$$

### 2.12. Particle Morphology and Particle Size Distribution

Particle morphology was evaluated by scanning electron microscopy (SEM) FEI Quanta 200 FEG which is a high resolution field emission scanning electron microscope with Schottky type electron gun where the samples can be investigated under controlled pressure water steam atmosphere. The SEM was operated at 10 kV with magnification of 5000–10,000 times. 

Particle size distribution was measured using laser light diffraction equipment Mastersizer 2000z, model Hydro 2000 MU (Malvern Instruments, Malvern, UK). A small sample of powder was suspended in ethanol 96% (*v/v*) under agitation, and the particle size distribution was monitored during each measurement until successive readings became constant. The surface-weighted and volume-weighted mean diameters were also measured. The relative span factor value, which describes the width of the particle size distribution, was calculated by equation:

(8) $$\mathrm{Span}=(d^{90}-d^{10})/d^{50}$$

where the values *d^10^*, *d^50^,* and *d^90^* give an indication of size of the fine (*d^10^)* and coarse (*d^90^*) fractions, and of the median particle size (*d^50^*). Smaller values of span indicate a narrower particle size distribution.

### 2.13. In Vitro Release Studies

Dissolution profiles of the active compounds rosmarinic acid and carvacrol in microcapsules were determined using a SOTAX brand AT7 smart model semi-automated dissolution tester (Switzerland) according to the procedure laid down in Ph.Eur. 8.7; 01/2016: 20903. Stomach-soluble gelatin capsules filled with oregano microcapsules were used for in vitro dissolution testing mimicking gastric conditions. The basket method was applied using artificial gastric juice without pepsin (AGJ, 50 rpm, 500 mL). The pH value was maintained at 1.5 (37 ± 0.5 °C). Gastro-resistant gelatin capsules filled with oregano microcapsules were used to mimic intestinal release for in vitro dissolution testing. The basket method was applied using 0.05 M phosphate buffer (pH 6.8). Aliquots (5 mL) were manually extracted from parallel dissolution vessels at 1, 3, 5, 7, 10, 15, 20, 25, and 30 min time points, filtered through a nitrocellulose membrane (0.45 µm) and quantified via HPLC. The dissolution media in each vessel was topped off with fresh dissolution fluid (5 mL) to restore the original volume. The mean value of six trial runs and a standard deviation were calculated. The evaluation of dissolution profiles was carried out in triplicate.

The in vitro release data were applied to the following kinetic models: zero order, first order, Higuchi, and Korsmeyer–Peppas to predict the mechanism and kinetics of rosmarinic acid and carvacrol release from microcapsules. The model that gave the highest correlation coefficient value was considered as the best fit of the release data [25].

### 2.14. Statistical Analysis

Data are presented as mean ± SEM. The software package Prism v. 5.04 (GraphPad Software Inc., La Jolla, CA, USA) was used to perform the analysis of variance (ANOVA) to detect differences among the mean values of responses and for the curve fitting. A value of *p* < 0.05 was taken as the level of significance.

## 3. Results and Discussion

The use of natural substances from plant sources has gained increasing popularity in alternative medicine mainly due to their potential to target oxidative and inflammatory processes at the root of many chronic disorders. However, herbal extract-based preparations are not stable due to oxidation and photolysis, and the low solubility of plant-based active compounds could impair their absorption in vivo [26,27,28]. Therefore, there is a challenge to find the optimal technological solutions to overcome these problems.

The object of our study was to optimize Turkish oregano extract microencapsulation by spray-drying to achieve high product yield and encapsulation efficiency of oregano main active compounds rosmarinic acid and carvacrol and narrower particle size distribution. During preliminary experiments, we have chosen gelatin as the main wall material for the microcapsules due to its ability to increase the emulsion viscosity and to form both film and gel, and supplemented it with gum Arabic, cyclodextrin β, and Tween 20 to obtain the best encapsulation efficiency.

### 3.1. Influence of Wall Material Component Amounts on the Encapsulation Efficiency of Rosmarinic Acid and Carvacrol

D-optimal experimental design using Design-Expert^®^ was applied to determine the optimal wall material composition. The effects of four independent variables: amounts of gelatin (A: 2–10 g), gum Arabic (B: 0–7.5 g), Tween 20 (C: 0–2 g), and β-cyclodextrin (D: 0–2 g) on two response variables: Encapsulation efficiency of rosmarinic acid and encapsulation efficiency of carvacrol were evaluated. The variables and their levels used in the design are presented in Table 1 (see Materials and Methods). The fitting models, equations, and statistical parameters are presented in Table 2.

**Figure 1:**
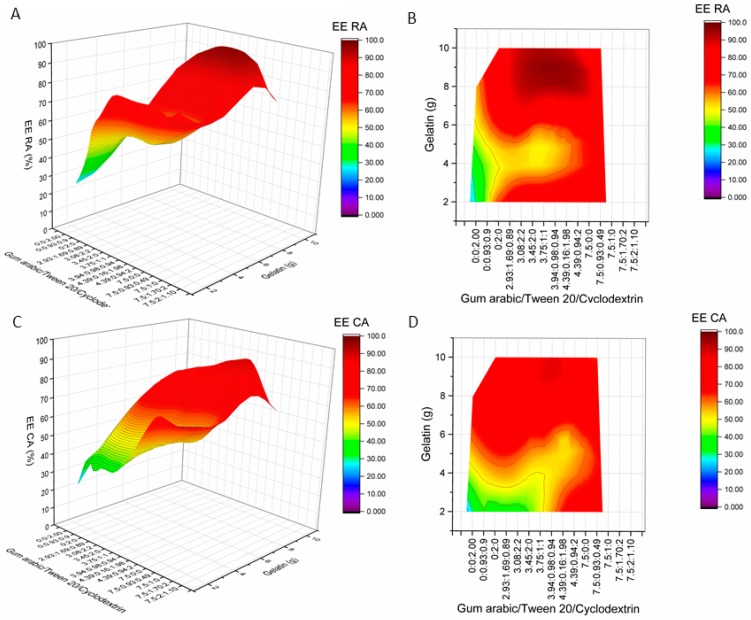
Influence of wall material component (gelatin supplemented with gum Arabic, Tween 20, and β-cyclodextrin) amounts on encapsulation efficiencies of rosmarinic acid (**A**,**B**) and carvacrol (**C**,**D**). RA—rosmarinic acid, CA—carvacrol, EE—encapsulation efficiency. (**A**,**C**)—surface plots; (**B**,**D**)—contour plots.

The responses—encapsulation efficiency of rosmarinic acid and encapsulation efficiency of carvacrol—were determined under 30 different experimental conditions (Figure 1).

**Figure 2:**
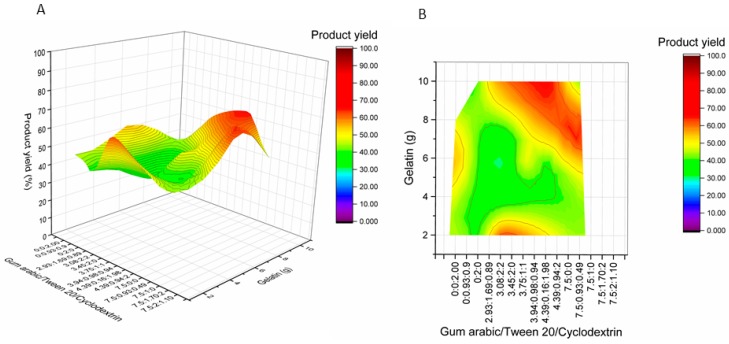
Influence of wall material component (gelatin supplemented with gum Arabic, Tween 20, and β-cyclodextrin) amounts on product yield of oregano microcapsules. RA—rosmarinic acid, CA—carvacrol, EE—encapsulation efficiency. (**A**)—surface plot, (**B**)—contour plot.

The spray-drying mixture consisted of two parts wall material solution and one part ethanolic oregano extract, the feed flow rate was 40 mL/min, and the air inlet temperature was 170 °C [8]. Product yields were also determined at 30 experimental conditions (Figure 2).

The highest encapsulation efficiencies of rosmarinic acid (99.6%) and of carvacrol (99.8%) were achieved when 10 g of gelatin, 4.3 g of gum Arabic, 0.94 g of Tween 20, and 2 g of β-cyclodextrin were used as wall materials in the emulsion. The highest product yield at these conditions was 80.65%.

Further, numerical optimization of wall material amounts using the desirability function has been performed. The optimization parameters are presented in Table 3.

The results showed that experimental values did not significantly differ from the predicted values (*p* > 0.05). The optimal composition of Turkish oregano microcapsules has been determined as follows: 10 g of gelatin, 7.5 g of gum Arabic, 1.99 g of Tween 20, 1.98 g of β-cyclodextrin, and 20 g of ethanolic oregano extract.

### 3.2. Physicochemical Properties of Optimal Formulation Microcapsules

**Figure 3:**
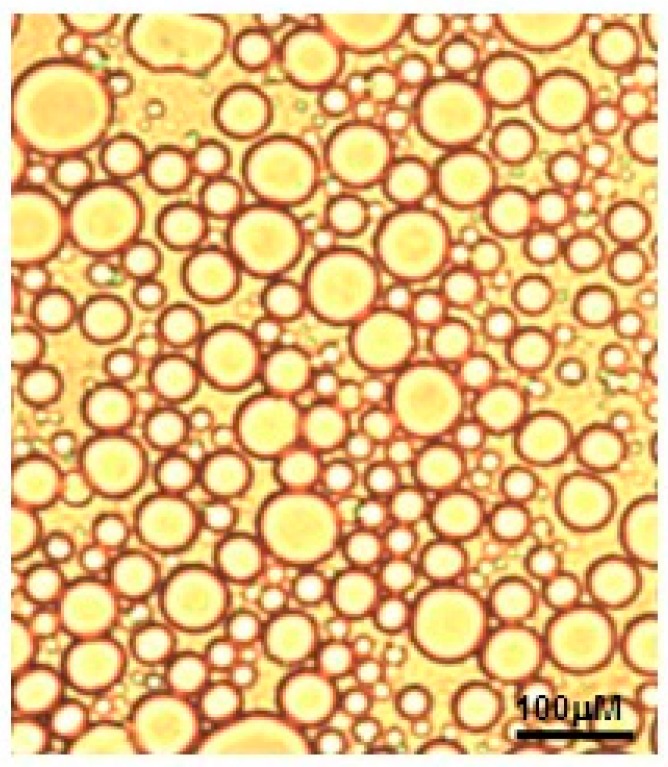
Optical microscopy of the optimized emulsion at 400× magnification.

Optical microscopy study revealed that the type of technologically optimized emulsion was oil-in-water, with droplet size of 45 ± 5 µm (Figure 3).

**Figure 4:**
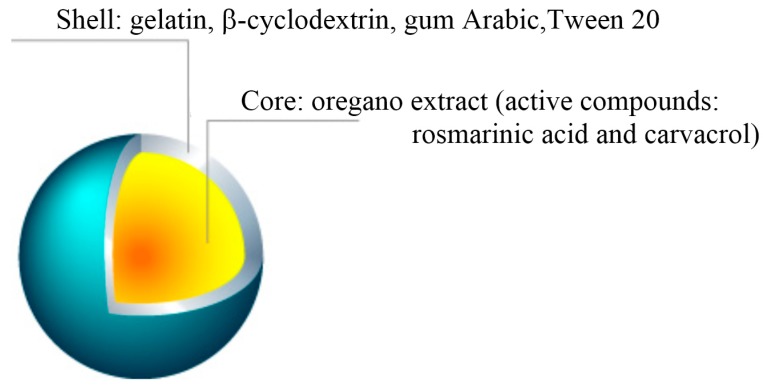
Microcapsule structure.

The viscosity of the emulsion at 100 rpm was 1034.17 ± 12.45 mPa/s. When the viscosity increases, the stability of the emulsion also increases [29]. The stability of the emulsions to creaming was assessed using a modified accelerated creaming test [30]. The obtained creaming index was equal to 0, thus the results showed that the emulsion is stable. The microcapsule structure is shown in Figure 4.

In the next experiments, the product yield, the encapsulation efficiencies, powder flowability, and moisture content of optimal formulation microcapsules were evaluated. The product yield and the encapsulation efficiencies depend on the properties of the wall material component solution used to prepare the emulsion for spray-drying [31]. During the process, the wall material components form the firm protective film around the formed particles. To protect the active compounds against the deteriorating effects of processing and storage conditions, as well as retard evaporation, microencapsulation has been considered as one of the most effective techniques. The results showed that the encapsulation efficiency of rosmarinic acid was 96.7%, the encapsulation efficiency of carvacrol was 99.8%, and the product yield was 85.63%. Similar product yields were obtained by other authors during encapsulation of oregano essential oils [7,18]. Thus, according to the obtained results, the best wall material composition has been selected in our study.

Since high moisture could lead to stickiness in the particles thus causing their agglutination and the deterioration of the encapsulated material [24], we determined moisture content in our microcapsules. The results showed that the moisture content was 3.14 ± 0.39% which corresponds to the requirements of the European Pharmacopoeia. Flow properties of microcapsules were evaluated according to Carr index and Hausner ratio [24]. Lower Carr index indicates better powder flowability, high Hausner ratio reflects lower capability of flowing freely. In our case, the Hausner ratio was 1.14 ± 0.24, Carr index—12.00 ± 2.3%, thus the produced microcapsules had good flowability.

**Figure 5:**
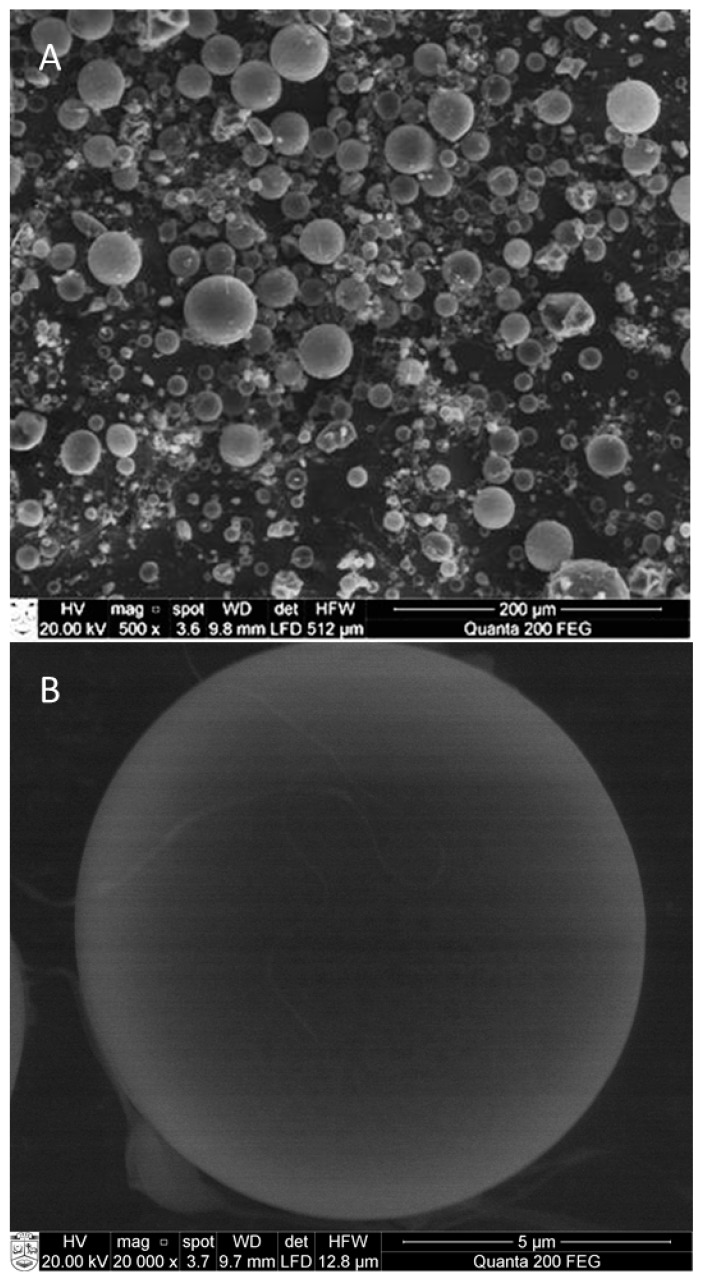
Scanning electron microscopy (SEM) of oregano microcapsules at lower (**A**) and higher (**B**) resolution. Microcapsule formulation: wall material (10 g of gelatin, 7.5 g of gum Arabic, 1.99 g of Tween 20, 1.98 g of β-cyclodextrin) and 20 g of ethanolic oregano extract as the core. Spray-drying parameters: The inlet temperature—170 °C, feed flow rate—40 mL/min, concentration of wall material in solution—20%.

The formation of microcapsules was verified by scanning electron microscopy (Figure 5).

All microparticles had uniform spherical shape and slightly dented surfaces. Shrinkage during the drying and cooling processes could be the reason of the irregular surface, and it is characteristic of the spray-drying process [7,32]. Obtained results are in good agreement with the observations from other authors [7,18].

**Figure 6:**
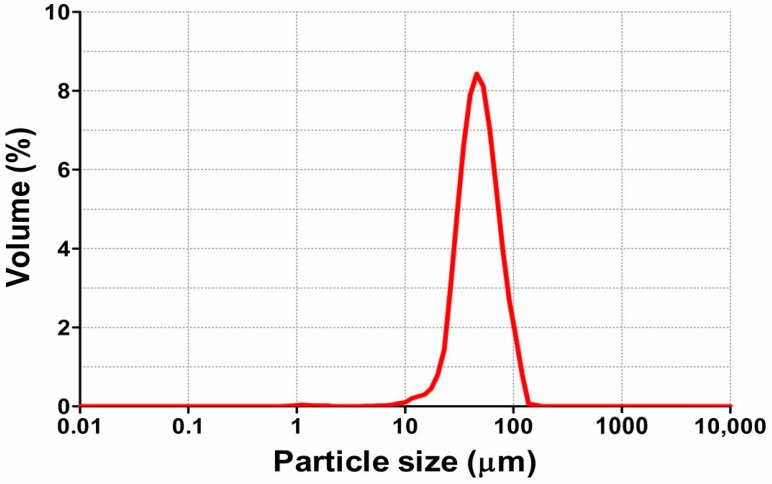
Particle size distribution of oregano microcapsules. Microcapsule formulation: wall material (10 g of gelatin, 7.5 g of gum Arabic, 1.99 g of Tween 20, 1.98 g of β-cyclodextrin) and 20 g of ethanolic oregano extract as the core. Spray-drying parameters: Inlet temperature—170 °C, feed flow rate—40 mL/min, concentration of wall material in solution—20%.

Particle size distribution was measured using laser light diffraction (Figure 6).

Particle size distribution and their uniformity index are very important technological characteristics responsible for the powder properties. In our study, the surface-weighted mean diameter was 50.96 ± 1.8 μm, and the calculated relative span factor value was 1.78. Thus, our results indicate the narrow particle distribution and the uniform system since the relative span factor is below 2 [32].

### 3.3. In Vitro Dissolution Studies

**Figure 7:**
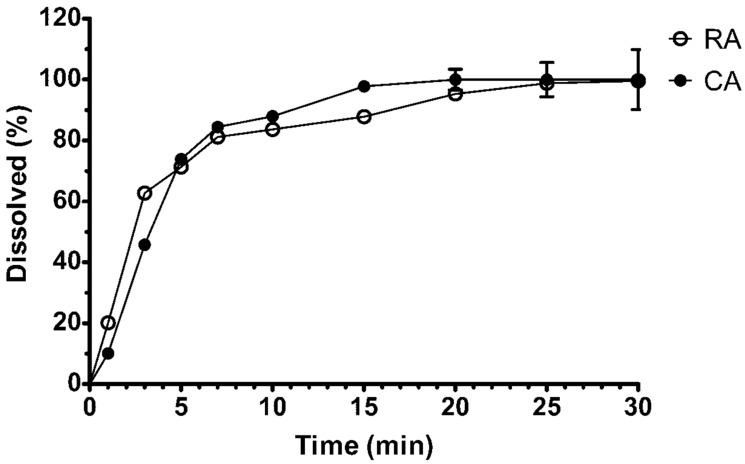
Release of rosmarinic acid (RA) and carvacrol (CA) in vitro from oregano microcapsules. Microcapsule formulation: wall material (10 g of gelatin, 7.5 g of gum Arabic, 1.99 g of Tween 20, 1.98 g of β-cyclodextrin) and 20 g of ethanolic oregano extract as the core. Spray-drying parameters: inlet temperature—170 °C, feed flow rate—40 mL/min, concentration of wall material in solution—20%.

It has been reported that the wall material composition could influence the bioavailability of active compounds in microcapsules [33,34]. Therefore, we investigated the in vitro release of rosmarinic acid and carvacrol from the optimal formulation of microcapsules in artificial gastric juice in our study. The in vitro dissolution profile of the prepared microcapsules is shown in Figure 5. 94.28 ± 0.46% of rosmarinic acid was released from the microcapsules after 20 min (Figure 7).

To mimic intestinal release, 0.05 M phosphate buffer (pH 6.8) and gastroresistant gelatin capsules filled with oregano microcapsules were used. The release profile was similar to that obtained with artificial gastric juice (Data not shown).

Fast rosmarinic acid release could be influenced by wall material components used in the preparation of microcapsules [35]. Carvacrol is poorly soluble in water together with hydrophilic active compounds [33], therefore one of the main purposes in our study was to improve its bioavailability. The results showed that 97.76 ± 0.4% of carvacrol was released after 15 min under our experimental conditions (Figure 7). This effect could be related to the wall material component β-cyclodextrin, which readily encapsulates hydrophobic active compounds and enhances their fast release and bioavailability [19,21,33]. 

Physicochemical properties of the active compounds as well as wall materials and the core: Wall material ratio influence the release of active compounds from the formulation and thus modify the release kinetics accordingly. The mathematical drug release models with major application and the best describing drug release phenomena are, in general, the Higuchi model, zero order model, first order model, and Korsmeyer–Peppas model [25]. Therefore, we applied these models to evaluate the kinetics and mechanisms of rosmarinic acid and carvacrol release from microcapsules (Table 4).

If active compound release follows zero order kinetics, the release rate is independent of active compound concentration, and is dependent on it if the release follows first order kinetics [25]. The Higuchi equation suggests active compound release by diffusion, whereas the Korsmeyer-Peppas power law equation defines the type of diffusion [25]. In our study, the release of rosmarinic acid and carvacrol depended on their concentration and was governed by diffusion (Table 4).

The stability investigations of the prepared microcapsules revealed that their physicochemical properties and content of rosmarinic acid and carvacrol remained unchanged for 12 months (data not shown).

## 4. Conclusions

During the study, the wall material components amounts were optimized for Turkish oregano microcapsules to improve the product yield, increase the encapsulation efficiency, and achieve narrower particle size distribution. When the wall material solution contained 10 g of gelatin, 7.5 g of gum Arabic, 1.99 g of Tween 20, 1.98 g of β-cyclodextrin, and 20 g of ethanolic oregano extract, the encapsulation efficiency of rosmarinic acid was 96.7%, the encapsulation efficiency of carvacrol was 99.8%, the product yield was 85.63%, and the particle size distribution was narrow (relative span factor value 1.78). The use of gelatin as the main coating material, in supplementation with gum Arabic, Tween 20, and β-cyclodextrin not only improved the encapsulation efficiency, but also increased the in vitro release of both main active compounds of Turkish oregano extract—rosmarinic acid and carvacrol.

## Figures and Tables

**Table 1:** D-optimal design with independent and response variables for the preparation of Turkish oregano-loaded microcapsules.

Run No.	Independent Variables				Response Variables	
	A	B	C	D	EE RA (%)	EE CA (%)
1	5.84	3.98	0.98	0.94	42.75	61.45
2	6.24	3.45	2.00	0	92.21	85.22
3	9.88	4.76	1.95	1.16	86.25	92.33
4	5.82	0	0.93	0.90	37.06	61.73
5	2.00	0	0	0	25.39	15.26
6	3.60	3.08	2.00	2.00	52.66	56.63
7	3.68	3.81	0.47	0	51.04	45.56
8	7.96	0	0	2.00	54.51	68.89
9	6.00	3.75	1.00	1.00	45.62	51.30
10	2.00	7.50	1.01	0	66.89	58.31
11	2.01	2.93	1.69	0.89	59.30	43.25
12	10.00	7.50	2.00	0.06	71.12	63.04
13	5.88	7.50	0	0	62.97	46.87
14	6.00	3.75	1.00	1.00	66.33	67.09
15	5.84	3.94	0.98	0.94	74.12	69.33
16	10.00	0	2.00	1.52	76.55	71.46
17	2.00	7.50	2.00	1.10	81.76	72.39
18	10.00	0	1.00	0	68.96	62.66
19	5.89	4.39	0.16	1.98	63.63	70.26
20	7.20	7.50	1.70	2.00	82.51	79.69
21	8.40	7.50	0.93	0.49	100.0	87.41
22	5.82	0	0.93	0.90	71.39	66.46
23	10.00	4.39	0.94	2.00	86.52	74.31
24	10.00	3.00	0	0	86.17	66.91
25	2.00	0	2.00	0	81.71	3.90
26	2.00	7.50	0	2.00	86.22	79.73
27	2.00	0	1.14	2.00	24.26	70.76
28	10.00	4.39	0.94	2.00	99.60	99.80
29	10.00	7.50	0	1.19	93.76	81.69
30	2.00	3.41	0	1.05	83.20	32.85

А—gelatin (g); B—gum Arabic (g); C—Tween 20 (g); D—β-cyclodextrin (g); EE RA—encapsulation efficiency of rosmarinic acid; EE CA—encapsulation efficiency of carvacrol.

**Table 2:** The fitting models, equations, and statistical parameters of the experimental design.

Response (%)	Min Value (%)	Max Value (%)	Model	*p* Value	r^2^	r^2^ Adjusted	r^2^ Predicted	Final Equation
EE RA	24.26	100	Linear	0.0001	0.9663	0.9242	0.9695	=+69.12 +10.49 A +12.43 B +3.59 C −0.000547 D
EE CA	3.9	99.8	Linear	0.0001	0.9827	0.9777	0.9765	=+64.03 +13.84 A +9.33 B +2.61 C +11.32 D

А—gelatin (g); B—gum Arabic (g); C—Tween 20 (g); D—β-cyclodextrin (g); EE RA—encapsulation efficiency of rosmarinic acid; EE CA—encapsulation efficiency of carvacrol.

**Table 3:** Numerical optimization of wall material amounts using desirability function.

**Independent Variables**			
	**Amount Levels (g)**	**Predicted Optimal Amount (g)**	
Gelatin (A)	2–10	10	
Gum Arabic (B)	0–7.5	7.5	
Tween 20 (C)	0–2	1.99	
B-cyclodextrin (D)	0–2	1.98	
**Response Variables**			
**Encapsulation Efficiency (EE)**	**Criteria**	**Predicted Mean Value (%)**	**Obtained Mean Value (%)**
Rosmarinic acid (RA)	Maximize	95.6	96.7
Carvacrol (CA)	Maximize	100.9	99.9

Desirability 0.971.

**Table 4:** In-vitro drug release profile application in different mathematical models.

Kinetic Model	Rosmarinic Acid			Carvacrol		
	r^2^ Value	Slope	Intercept	r^2^ Value	Slope	Intercept
Zero order	0.68	5.35	26.73	0.79	6.58	18.52
First order	0.87	−0.06	1.86	0.98	−0.11	2.03
Higuchi	0.90	24.77	6.25	0.92	28.80	−3.17
Korsmeyer–Peppas	0.91	70.44	15.85	0.97	83.27	7.16
